# Condensation methodology for quantification of Polymyxin B fluorimetrically: application to pharmaceutical formulations and greenness assessment

**DOI:** 10.1186/s13065-024-01156-9

**Published:** 2024-05-29

**Authors:** Mahmoud A. Abdelmajed, Khalid M. Badr El-Din, Tamer Z. Attia, Mohamed Oraby, Mahmoud A. Omar

**Affiliations:** 1 Department of Pharmaceutical Analytical Chemistry, Faculty of Pharmacy, Deraya University, New Minia, Egypt; 2 Department of Analytical Chemistry, Faculty of Pharmacy, Minia University, Minia, Egypt; 3 Department of Pharmaceutical Analytical Chemistry, Faculty of Pharmacy, Sohag University, Sohag, Egypt; 4 Department of Pharmacognosy and Pharmaceutical Chemistry, College of Pharmacy, Taibah University, Medinah, Saudi Arabia

**Keywords:** Polymyxin B, Ninhydrin, Phenylacetaldehyde, Ophthalmic and intravenous pharmaceutical forms, Methodology’s greenness

## Abstract

The appearance of multidrug-resistant Gram-negative bacterial infections, along with the lack of newly discovered antibiotics, resulted in the return to old antimicrobial medications like Polymyxins. As a result, the suggested technique aims to develop a fast, environmentally friendly, and sensitive fluorimetric method for quantifying Polymyxin B. The investigated approach depends on generating a highly fluorescent derivative by a condensation pathway between the studied drug and ninhydrin in the presence of phenylacetaldehyde and then estimated spectrofluorimetrically. After the reaction conditions were well optimized, the fluorescent product was estimated at emission wavelength (λ_em_) = 475.5 nm (following excitation at a wavelength (λ_ex_) = 386 nm. The developed calibration plot displayed rectilinear throughout the following range (0.2-3 µg mL^− 1^), and the calculated limit of detection and quantification were 0.062 µg mL^− 1^ and 0.187 µg mL^− 1^, respectively. As a consequence, the drug’s ophthalmic and intravenous pharmaceutical forms were both successfully quantified with an excellent degree of recovery. Finally, the methodology’s greenness was assessed utilizing Analytical Eco-Scale scores.

## Introduction

**Fig. 1:**
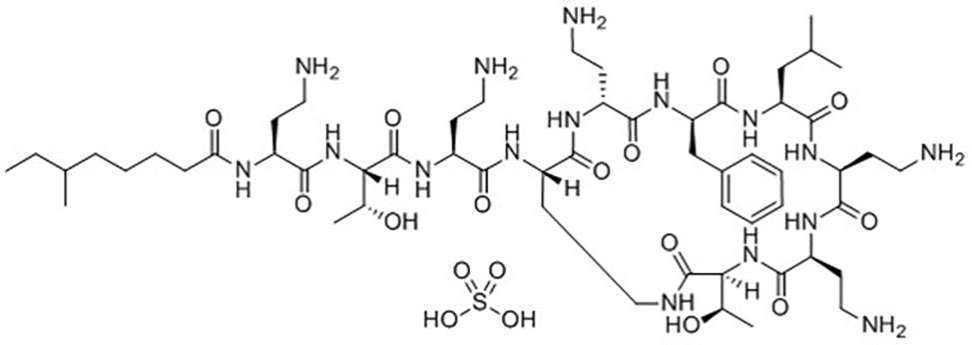
Polymyxin B sulfate (Poly B) chemical structure

The treatment of multidrug-resistant (MDR) bacterial infections remains a major unresolved clinical demand despite considerable progress across the scientific fields. Due to the limited development of new antibiotics capable of treating these types of infections, scientists are returning to older antimicrobial medications [1]. Polymyxins (PMS), notably Polymyxin B (Poly B), have attracted attention due to their specific efficacy against MDR Gram-negative bacteria. Poly B, a cyclic polypeptide protein widely used for clinical purposes, disrupts bacterial cell membranes, causing cell death, as shown in (Fig. 1).

Belongs to published papers, more than one chromatographic approach as HPLC [2–7] and LC-Mass [8–19] also microbiological articles were reported [20, 21]. On the other hand, spectroscopic techniques were listed, including spectrophotometric [22–25] and spectrofluorimetric [26–28] approaches. Owing to its simplicity, sensitivity, and no need for a complex apparatus or sample preparation, the spectrofluorimetry technique is frequently employed in recent drug analyses. On the other side, separative techniques have several drawbacks such as excessive solvent utilization, equipment with high cost, and exhausted extraction operations. Furthermore, spectrophotometric and microbiological tests lack sensitivity.

The previously reported fluorimetric methods had limitations such as utilizing drastic conditions (boiling for a long time, 35 min.) [28] or employing an expensive reagent [27], or using hazardous chemicals [26]. So the described approach aimed to overcome these drawbacks by establishing a fast, cost-effective, and environmentally friendly methodology to be easily applied in routine quality control assay.

Ninhydrin, in the presence of phenylacetaldehyde, is a commonly used derivatizing reagent for numerous primary amine-containing pharmaceuticals for spectrophotometric and spectrofluorimetric analysis, owing to its accuracy, cost-effectiveness, and reproducibility. In this study, the fluorogenic reagents interacted with Poly B’s primary amine groups to form a highly fluorescent derivative, which was detected at λ_ex_ = 386 nm and λ_em_ = 475.5 nm.

## Experimental

### Devices

A Jasco FP-8350 spectrofluorimeter (Tokyo, Japan) was employed to obtain the spectrofluorimetric measurements. The apparatus had a 150 W Xenon-arc lamp, a 400 V PMT voltage, a 5 nm slit width for both the emission and excitation monochromators, and a 1000 nm. min^− 1^ scan rate. Also, a temperature controller (Schwabach, Germany) and pH meter Adwa AD 1100 (Romania) were utilized in the prescribed approach.

### Chemicals and reagents

Standard Poly B was kindly gifted from The National Organization of Drug Control and Research (NODCAR), which was used without additional purification. Paximid® vial a product of Cipla Company product (is claimed to contain 500,000 IU, which is equal to 5 mg of the studied drug). Terramycin® ophthalmic ointment, a product from Pfizer pharmaceutical company (contains 10,000 IU of Poly B equivalent to 1.6667 mg per 1 gm). Ninhydrin, 0.1% (w/v) (Alpha Chemicka, Mumbai, India) was daily prepared in distilled water, and phenylacetaldehyde, 0.2% (v/v) (Sigma Aldrich, Germany) was weekly set in ethyl alcohol. All experimental solvents, including acetone, acetonitrile, methyl alcohol, ethyl alcohol, hexane, and dimethylformamide (DMF), were supplied by ElNasr Chemical CO (Cairo, Egypt). The same company also supplied phosphoric acid, citric acid, HCL, and NaOH. Adjust the pH of Teorell & Steinhagen buffer using 0.1 M HCL after mixing suitable quantities of 1 molar sodium hydroxide, citric, and phosphoric acids.

### Standard drug solution preparation

The Poly B stock solution at a final concentration of 100 µg mL^− 1^ was daily prepared by dissolving 10 mg of Poly B in distilled water. After further dilution, working solutions were obtained.

### General analytical procedures

Numerous test tubes were pipetted with 1 mL of working solutions in a range of 2 to 30 µg mL^− 1^ along with 1 mL of Teorell& Steinhagen buffer (pH = 7), and 1 mL of 0.1% (w/v) ninhydrin, and 0.02% (v/v) phenylacetaldehyde solutions. To ensure a reaction pathway was completed, all tubes were kept in a water bath of 80 °C for 15 min, after which it was cooled in an ice bath. The mixtures in the test tubes were transferred to 10.0-mL volumetric flasks and completed to mark using ethyl alcohol. Lastly, relative fluorescence intensity (RFI) was evaluated at λ_em_ = 475.5 nm (after λ_ex_ = 386 nm). With each experiment, a blank was employed, following all previous procedures but without the addition of the studied drug.

### Preparation of vial solution

An accurate amount from Paximid® vials equal to 10 mg of Poly B was transferred to a 100-mL volumetric flask and completed to mark with the employed solvent. Sequential dilution was carried out until the concentration reached (2–30 µg mL^− 1^), after which all steps in Sect. 2.4 were repeated.

### Preparation of ophthalmic ointment solution

An exact quantity of Terramycin® eye ointment equivalent to 10 mg of Poly B was vigorously shaken with 30 mL of a mixture solution of distilled water and n-hexane in a ratio of 1:1. Using a separating funnel, the two immiscible layers were obtained, after which the aqueous layer was collected and the organic one received further aqueous washings. Then the collecting solution was transferred to a 100-mL volumetric flask and completed to mark by distilled water. After filtering, further dilution was employed to obtain working solutions, and general analytical procedures were followed.

## Results and discussion

**Fig. 2:**
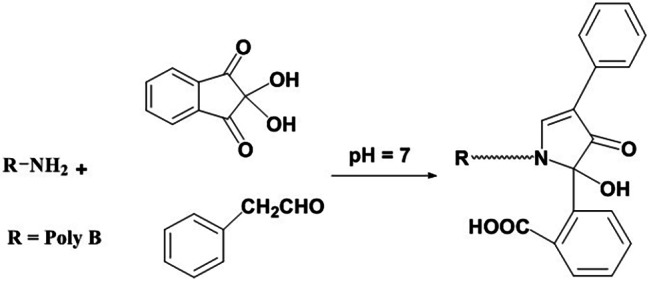
Suggested pathway of the reaction between the investigated drug and the fluorogenic reagent

**Fig. 3:**
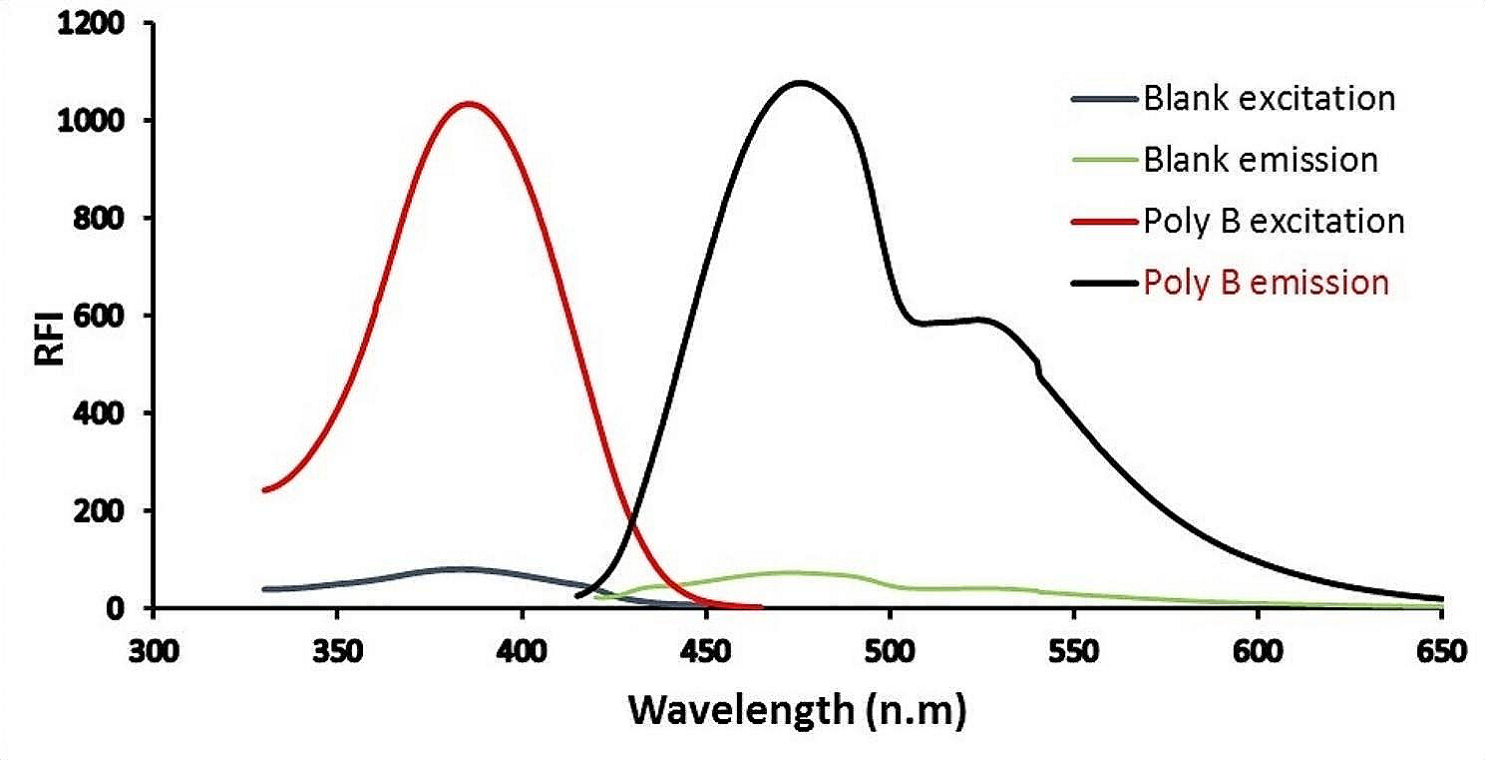
Excitation and emission spectra of Poly B (2 µg mL^− 1^) with fluorogenic reagents

In the presence of phenylacetaldehyde, ninhydrin is utilized as a derivatizing agent to assay drugs with primary amine groups. producing yellow fluorescent derivatives [29–36]. In the developed experiment, ninhydrin condensed with the amino moiety of Poly B in addition to phenylacetaldehyde at λ_em_ = 475.5 nm (following excitation at λ_ex_. = 386 nm). The reaction pathway and the spectra are illustrated in (Fig. 2) and (Fig. 3), respectively. To raise the value of the current work, a comparative table with other reported fluorimetric articles was established, and all values were inserted in (Table 1).

**Table 1:** A comparison between the investigated study and the reported fluorimetric methods

Method	Linear range (µg mL^− 1^)	Temperature	Cost	Hazardous agents	Ref.
Hantzsch Reaction	0.1–3	100 ºC (boiling) for 35 min.	Cost-effective	Not utilized	[28]
NBD-CL Reaction	0.1–1.2	60 ºC for 45 min.	Not cost-effective	Conc. H_2_SO_4_	[26]
Fluorescamine Reaction	0.07–1.8	Room temperature for 10 min.	Not cost-effective	Not utilized	[27]
Ninhydrin & phenylacetaldehyde	0.2–3	80 ºC for 15 min.	Cost-effective	Not utilized	Current work

### Optimization of the reaction parameters

To determine the ideal reaction conditions that would yield the highest RFI values, each parameter was optimized while the others remained fixed.

#### Buffer optimization

**Fig. 4:**
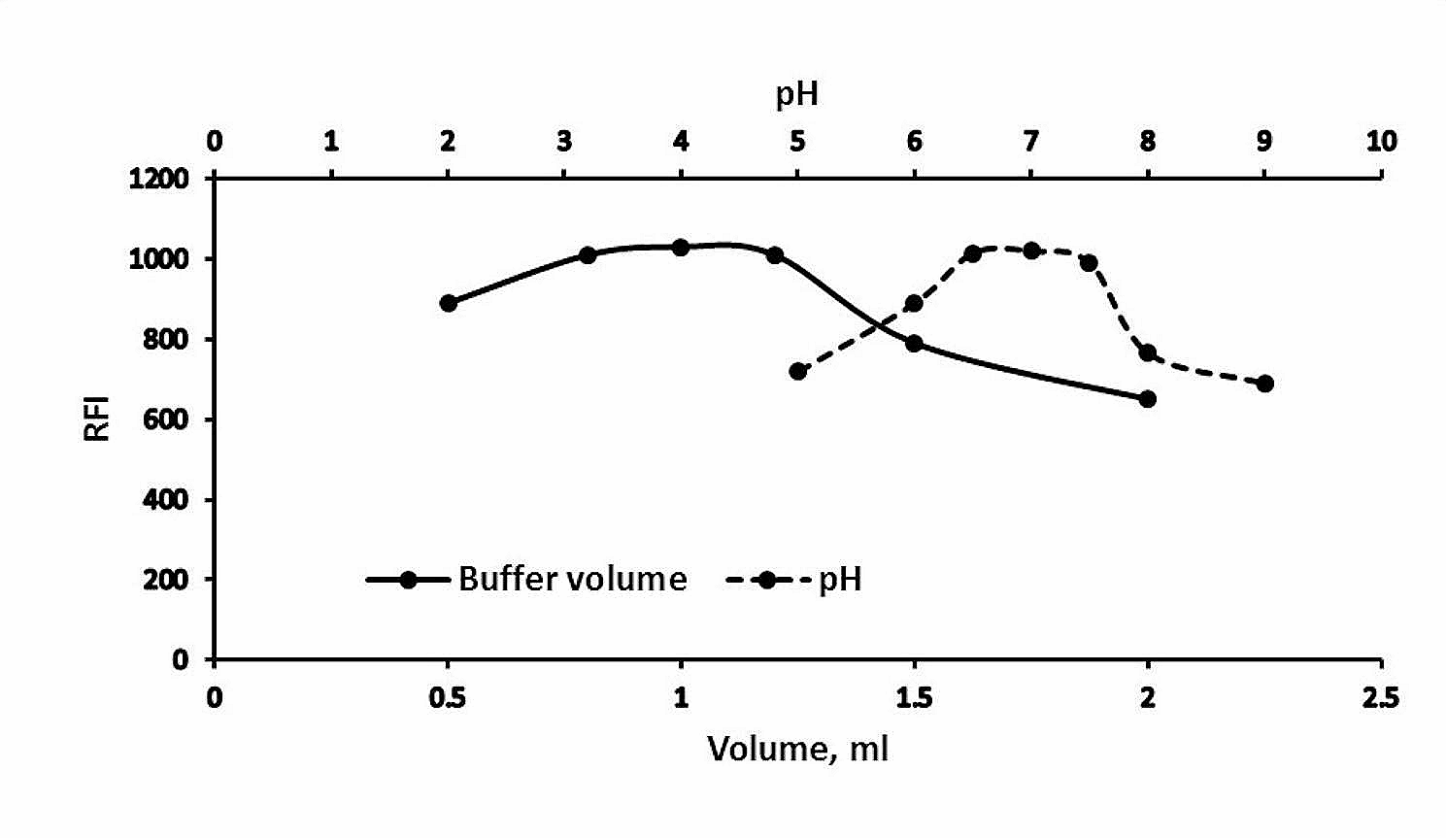
Effect of the pH and buffer volume on the RFI of the reaction product of Poly B (2 µg mL^− 1^)

Since any small change in the pH of the experimental media had a considerable influence on the approach’s fluorescence intensity, Teorell & Steinhagen buffer was employed to establish a pH scale (5–9). The RFI peaked at a pH range (6.8–7.2); any deviation led to a significant decrease in fluorescence intensity. While checking the optimal buffer volume, the (0.8–1.2 mL) range yielded the best outcomes. So, one mL of the utilized buffer (pH 7) was the best choice. Data are shown in (Fig. 4).

#### Regeants volume optimization

**Fig. 5:**
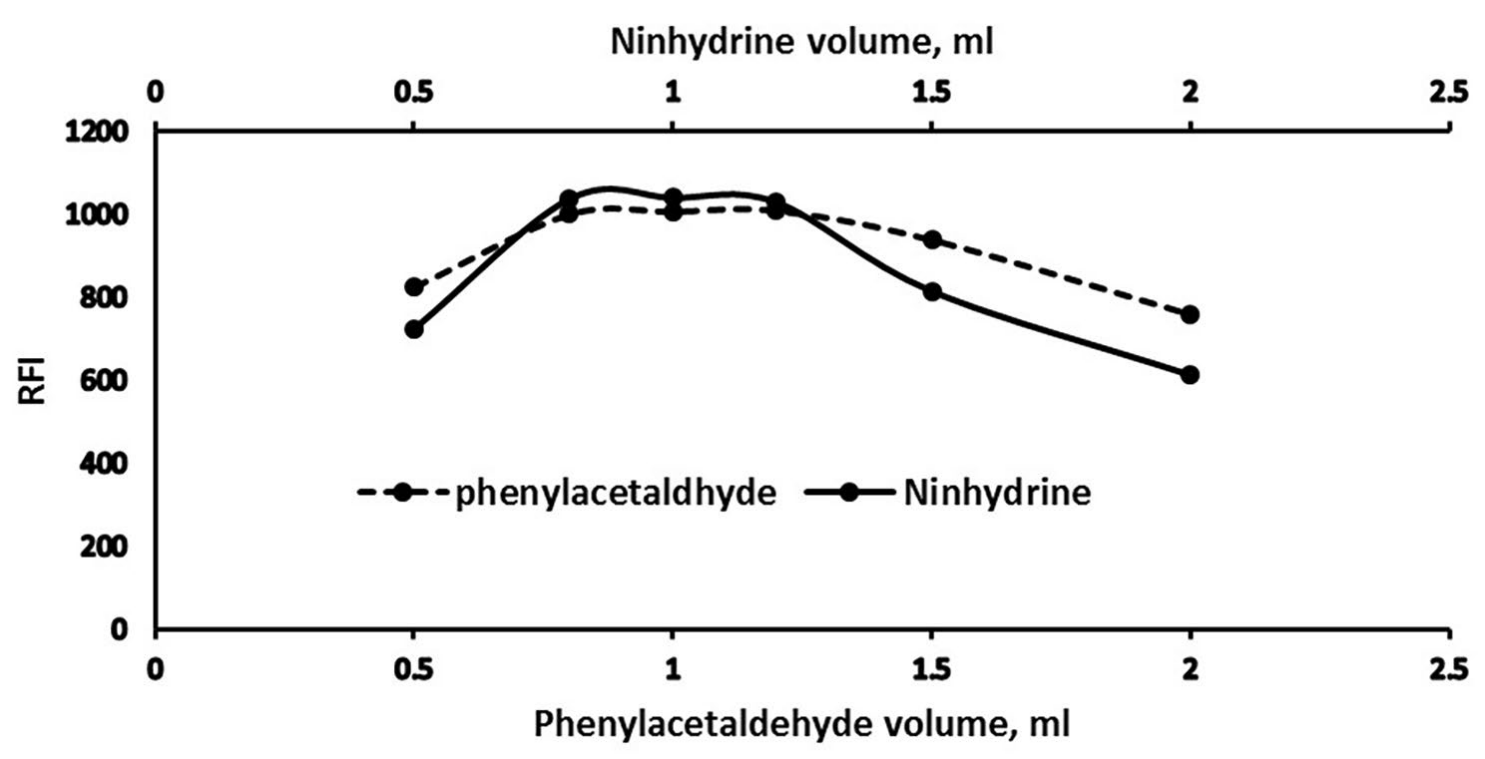
Effect of fluorogenic reagents volumes on the RFI of the reaction product of Poly B (2 µg mL^− 1^)

To study the optimum volumes of ninhydrin and phenylacetaldehyde affect the approach performance, a scale of (0.5–2.0 mL) was successfully investigated. RFI was raised in tandem with increased volumes of each of them until a plateau was reached at 0.8 to 1.2 mL, beyond this range, fluorescence declined. Therefore, 1.0 mL was the optimum volume from each reagent. All information was gathered in (Fig. 5).

#### Temperature and heating time optimization

**Fig. 6:**
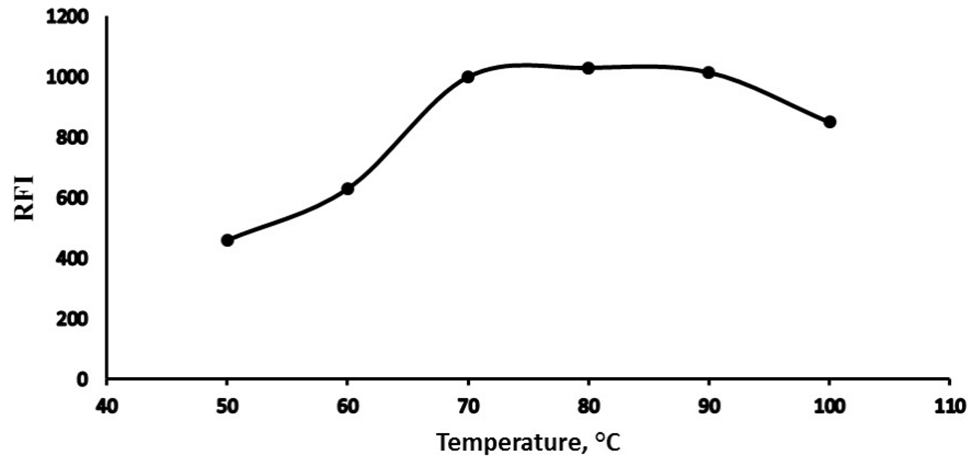
Temperature on the RFI of the reaction product of Poly B (2 µg mL^− 1^)

**Fig. 7:**
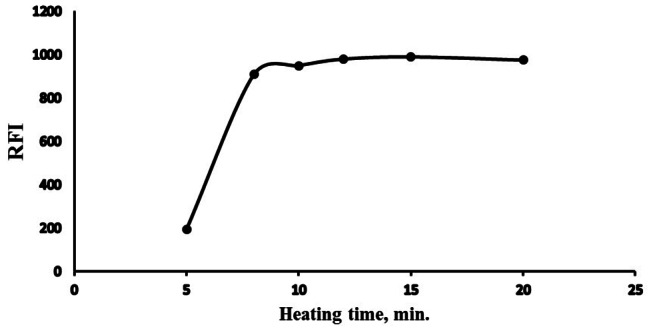
Heating time effect on the RFI of the reaction product of Poly B (2 µg mL^− 1^)

A temperature controller instrument was employed to get various degrees of temperatures in the range of 50 to 100 °C. RFI peaked at 60 to 90 °C. After which, various heating time was tested at 80 °C. Fluorescence increased rapidly as the time intervals were extended until reached a steady line at 10 to 20 min. As a result, heating at 80 °C for 15 min. was the optimum thermal setting. All data were gathered in (Fig. 6) and (Fig. 7).

#### Solvent optimization

**Fig. 8:**
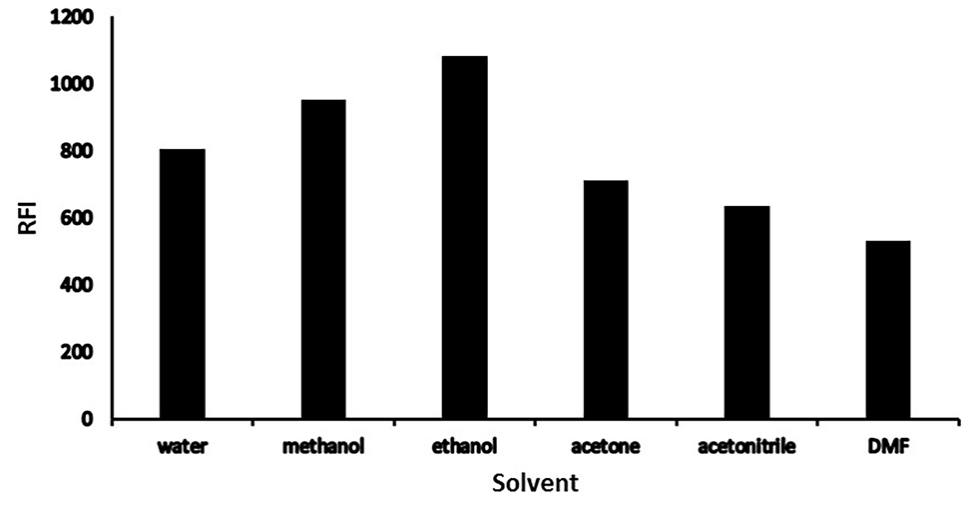
Solvent type effect on the RFI of the reaction product of Poly B (2 µg mL^− 1^)

While other variables were kept constant, acetone, acetonitrile, ethyl alcohol, methyl alcohol, distilled water, and DMF were employed to further dilute the final fluorescent products. Ethyl and methyl alcohols have the greatest RFI levels, consequently, ethyl alcohol was chosen as the optimum one owing to its greenness profile, as displayed in (Fig. 8).

### Validation of the studied method

The proposed approach was carefully validated using ICH criteria [37], which included estimating its linearity and range, accuracy, precision, robustness, and sensitivity, as well as LOD and LOQ.

#### Linearity & range

A calibration curve of the developed method was plotted of Poly B different concentrations versus RFI values. A linear relationship in the range of 0.2 to 3 µg mL^− 1^ was obtained, with a correlation coefficient value of 0.9996. All analytical parameters related to the linear regression equation were inserted in (Table 2).

**Table 2:** Regression equation and related validation parameters

Parameters	Investigated approach
λ_ex_ (nm)	386
λ_em_ (nm)	475.5
Linear range (µg mL^− 1^)	0.2–3
Correlation coefficient (r)	0.9996
Determination coefficient (r^2^)	0.9993
Intercept ± SD*	-71.72 ± 9.274
Slope ± SD	497.13 ± 5.99
Calculated LOD (µg mL^− 1^) **	0.062
Calculated LOQ (µg mL^− 1^) ***	0.187

* **SD**: Standard Deviation

**LOD: Limit of detection

***LOQ: Limit of quantitation

#### Accuracy

Over the methodology’s calibration range, five different concentrations of the studied drug of 0.2, 0.6, 1, 2, or 3 µg mL^− 1^ were evaluated three times. The calculated data showed a significant agreement between the experimental and true values, proving the current technique was accurate. All values were inserted in (Table 3).

**Table 3:** Evaluation of the accuracy of the proposed spectrofluorimetric method

Sample no.	Taken conc. (µg mL^− 1^)	Found conc. (µg mL^− 1^)	% R*
1	0.2	0.199	99.50
2	0.6	0.589	98.17
3	1	1.018	101.80
4	2	1.987	99.35
5	3	3.009	100.30
Mean			99.82
SD			1.341
RSD			1.343

RSD: relative standard deviation *Mean of three replicate measurements

#### Precision

Three different drug concentration levels (0.6, 1.5, and 3 µg mL^− 1^) and three duplicates of each one were utilized throughout the day and over three successive days to assess intra- and inter-day precision, respectively. The calculated mean relative standard deviation (RSD), did not surpass 2, indicating that the proposed methodology was reliable and reproducible. (Table 4) gathering all information.

**Table 4:** Developed approach intra- and inter-day precisions

Concentration level (µg mL^− 1^)	% R* ± RSD	
	Intra-day	Inter-day
0.6	98.98 ± 0.88	101.21 ± 0.72
1.5	100.12 ± 1.76	99.66 ± 1.26
3	99.54 ± 1.33	100.09 ± 1.35

* Mean of three determinations

#### Robustness

The method’s robustness was estimated by checking small changes in approach conditions like pH and fluorogenic reagent volumes. The tested parameter was altered while the others remained constant, then the recovery percentage (%R) was estimated. Minor alterations had no significant influence on the approach’s performance since the obtained RSD did not exceed 2%, so the outcomes confirmed the robustness of the described approach. All values were inserted in (Table 5).

**Table 5:** Robustness for determination of Poly B (2 µg mL− 1) by the developed approach

Method Parameters	% R* ± SD	RSD
pH		
6.8	98.69 ± 0.38	0.39
7	100.72 ± 1.15	1.14
7.2	99.19 ± 0.70	0.71
Volume of Ninhydrin		
0.8	99.72 ± 0.78	0.78
1	101.06 ± 1.65	1.63
1.2	100.33 ± 0.44	0.44
Volume of Phenyl acetaldehyde		
0.8	100.80 ± 1.22	1.21
1	99.97 ± 0.47	0.47
1.2	98.99 ± 1.03	1.04

* Mean of three determinations

To assess the sensitivity of the technique under investigation, LOD, and LOQ values were computed. Using the equation “LOD = 3.3 × ϭ / S or LOQ = 10 × ϭ / S,” where S denotes the calibration graph’s slope and ϭ denotes the intercept’s standard deviation, LOD = 0.187 µg mL^− 1^ and LOQ = 0.062 µg mL^− 1^ were accurately estimated.

### Application of the developed method

The suggested methodology was successfully employed to determine the amount of Poly B in the paximid® vial and Terramycin® eye ointment. %R values of the established approach were statistically compared with a previously published article [28], which showed lower T and F values than tabulated ones, showing good accuracy and precision of the developed methodology. All data were observed in (Table 6).

**Table 6:** Data for quantification of pharmaceutical formulations by the investigated method and compared with the reported one [28]

Dosage form	Mean of % R^*^ ± SD		*t*-value^#^	*F*-value^#^
	investigated approach	Comparison method		
Paximid® vial	99.95 ± 1.39	99.53 ± 0.56	0.485	6.146
Terramycin® eye ointment	99.71 ± 0.57	98.99 ± 0.83	0.962	5.88

^*^Mean is average of five determinations

^*^Tabulated values at 95% confidence limit are *t-* value = 2.306, *F-* value = 6.338

### Greenness evaluation

Various metrics have been used to assess the analytical method’s greenness, such as Analytical Eco-Scale (AES) [38]. The formula used to compute the AES score is 100 minus the total penalty point, considering parameters such as reagent amounts, occupational risks, waste, and energy. The higher the score reflects the more green the methodology. The designed approach included no extraction step, and the operation consumed less than 0.1 kW/h of energy for one sample. With an Eco-Scale score of 90, the developed approach can be regarded as having outstanding greenness. A comparison between the developed and reported approaches is illustrated in (Table 7).

**Table 7:** Comparison between the prescribed and reported method for assessing the approach’s greenness according to Analytical Eco-Scale tool

Item		The prescribed method			The reported method		
		Parameter	Word sign	Penalty point	Parameter	Word sign	Penalty point
Reagent	Amount		< 10 mL	0		< 10 mL	0
	Hazard	Ninhydrin	LSH**	1	Acetylacetone	LSH	1
		Phenylacetaldehyde	LSH	1	Formaldehyde	LSH	1
		Ethanol	None	0	Distilled Water	None	0
		Hexane	MSH*	2	Hexane	MSH	2
					Acetonitrile	LSH	1
Instrument	Energy (Spectrofluorimetry)	≤ 0.1		0	≤ 0.1		0
	Heater	Temperature (80 °C for 15 min)		1	Temperature (100 °C for 35 min)		3
	Occupational hazard	Emission of vapors and gases to the air		3	Emission of vapors and gases to the air		3
	Waste	1–10 mL		3	1–10 mL		3
TPPs***				11			14
Eco-Scale score	= 100 - TPP			89			86

MSH* More Severe Hazard LSH

** Less Severe Hazard TTPs

*** Total Penalty Points

## Conclusion

Through utilizing the amino group present in Poly B, designing a new and rapid fluorimetric strategy for quantification of the cited drug in intravenous and ophthalmic dosage forms was the aim of the current work. The extraction step, as well as the employment of instruments with a high cost, solvents, and reagents with a high grade of purity, were all obstacles that were overcome in the current approach. Lastly, AES tool was employed to assess the approach’s greenness. As a consequence, the current methodology could successfully quantify the studied drug in quality control laboratories with a high degree of reproducibility and greenness.

## Data Availability

The datasets during and/or analyzed during the current study are available from the corresponding author upon reasonable request.
